# Perceptions of student accoucheurs regarding gender inequality in midwifery training at Free State maternal healthcare institutions

**DOI:** 10.4102/curationis.v44i1.1988

**Published:** 2021-02-18

**Authors:** Siphiwe T. Madlala, Thembelihle S. Ngxongo, Maureen N. Sibiya

**Affiliations:** 1 Department of Nursing Science, Faculty of Science and Agriculture, University of Zululand, Kwadlangezwa, South Africa; 2 Department of Nursing Science, Faculty of Health Sciences, Durban University of Technology, Ritson, South Africa

**Keywords:** gender inequality, maternal healthcare, perceptions, pregnant women, student accoucheurs

## Abstract

**Background:**

Worldwide, gender inequality has been a dominating factor in the training of student accoucheurs in most maternal healthcare institutions. This poses challenges for the maternal healthcare institutions where student accoucheurs are placed for clinical practice as most women become reluctant or refuse to accept their services. Gender inequality has a negative impact on the training of student accoucheurs as most of them become demotivated which could lead to a high attrition rate.

**Objectives:**

This study explored and described the perceptions of student accoucheurs regarding gender inequality in midwifery training at the Free State maternal healthcare institutions.

**Method:**

An explorative and descriptive qualitative research design was undertaken. There were 40 student accoucheurs that formed 10 focus group discussions. Each focus group discussion had four participants. Tesch’s eight-steps of data analysis was used to analyse data.

**Results:**

Three major themes emerged during data analysis: meeting the training requirements in midwifery, women’s autonomy in a choice of healthcare provider versus student accoucheurs’ autonomy to be trained in midwifery and staff establishment to render maternal healthcare.

**Conclusion:**

The participants perceived prejudice, rejection and resistance by women in maternity units as a contributing factor to gender inequality, which has a negative impact on their training in midwifery. The study recommends that health directorates, nurse managers and training institutions should consider revising maternal healthcare policies regarding the recruitment and placement of willing accoucheurs in maternity units in order to address gender inequality.

## Introduction and background of the study

Over the past years, nursing has seen a steady change in the admission and training of student nurses, particularly males, in midwifery discipline. Despite the changing era, male nurses account for a small minority of the nursing profession, primarily because nursing is traditionally and predominantly considered a female profession (Cottingham 2014:133). The nursing role of caring has been largely equated to the feminine attributes; as a result, men in nursing are seen as misplaced and therefore faced with barriers (Achora 2016:24). However, the increasing healthcare needs, the global crisis in nursing shortage and the need for job security have led to more and more men joining the profession (Achora 2016:24). Florence Nightingale regarded women as carers and regarded it as a natural phenomenon for women to care for the sick; as a result, the expectations of caring and being gentle are now more often aligned with women than with men (McWilliams, Schmidt & Bleich 2013:38). Therefore, those barriers include, amongst others, the gender stereotype that men are not allowed to practise midwifery in some institutions. This stereotype is because of cultural and religious beliefs of pregnant women that men are not allowed in the maternity units. These beliefs lead to rejection and discrimination of student accoucheurs during their clinical placement in maternity units (Achora 2016:25). According to Ndou and Moloko-Phiri (2018:1), although over the last 20 years more men have chosen nursing as a profession, they remain a minority in most parts of the world, including South Africa. Stokowski (2012:77) revealed that male registered nurses and accoucheurs comprise less than 10% of the registered nurses in the United States of America, whereas in England 9 out of 10 nurses are women, 9 out of 8 midwives are women and 3 out of 4 supervisors are women. In the United Kingdom, gender inequality in nursing is still problematic as 91% of midwives are women and less than 2% are accoucheurs (Ira, Ellis & McFarlane 2014:516; Kouta & Kaite 2011:59). In the same vein, accoucheurs are still a minority in South Africa. The South African Nursing Council (SANC) Statistics, 2/2018 revealed that there were 94 657 midwives in comparison to 9594 accoucheurs registered in the roll. Furthermore, the SANC Statistics, 2/2018 revealed that in the Free State province, there were 1162 registered male nurses in the SANC roll compared to 7020 registered female nurses. Based on these numbers, it is evident that there are less accoucheurs than midwives.

Midwifery nursing is a highly gendered profession with characteristics of intimate care provision, nurturing and emotional support contributing to its ‘feminisation’ (Jafree, Zakar & Zakar 2014:1). This proves that despite the ongoing training of nurses worldwide, few male nurses join the nursing profession and even fewer practise midwifery on completion of training. Furthermore, Jafree et al. (2014:2) confirmed that midwifery nursing is a female-dominated profession; hence, high gender inequality in the training of student accoucheurs prevails. Gender inequality in maternity units does not affect the training of student accoucheurs in South Africa only but in most parts of the world too. Although no drastic measures have taken place in the South African maternal health regarding the training of student accoucheurs, it became evident in other countries that gender has a negative impact on the training of student accoucheurs. A study conducted by Bwalya et al. (2015:44) revealed that gender in midwifery nursing affected the training of student accoucheurs in Fiwale and Mpongwe Missionary hospitals, both situated in Ndola area where student accoucheurs were ordered to stop conducting deliveries by Chief Besa and Chief Mushili. Furthermore, in Zambia, student accoucheurs were banned from conducting deliveries in a local hospital by the hospital management following reports that pregnant women were shunning health institutions with student accoucheurs (Chasowa et al. 2015:69). Pregnancy and childbirth in this country are viewed as strictly a female domain. Similarly, in Zimbabwe, most women are culturally and traditionally sensitive during pregnancy, and they fear being attended by student accoucheurs which prevents them from attending antenatal clinics and hospitals where student accoucheurs are placed (Chasowa et al. 2015:74). In most cases, the gender of student accoucheurs has a negative impact on their training in maternal healthcare institutions, leading to increased attrition rates as compared to their female counterparts (McLaughlin, Muldoon & Moutray 2010:303). In 2017, the Free State School of Nursing had 138 female student nurses registered in their final year midwifery nursing science and 55 male student nurses in the same class. There were 123 female student nurses who passed their midwifery discipline and 35 student accoucheurs who passed their midwifery nursing science in the same year (SANC Statistics 2017:1). Therefore, it is evident that fewer student accoucheurs complete their training successfully in the midwifery discipline, which might be attributed to various barriers. Despite the successful throughput of midwives and accoucheurs at the training institutions, the number of student accoucheurs completing their midwifery training remains relatively low compared to female student midwives in the Free State province.

The fewer numbers of accoucheurs trained in midwifery nursing may also have a negative impact on male students who are currently enrolled in midwifery nursing as they lack male role models and mentors in midwifery clinical settings. Achora (2016:24) supported this notion by stating that the lack of accoucheur role models to mentor student accoucheurs in clinical areas leads to attrition of student accoucheurs in midwifery nursing. Furthermore, most nurse managers in the maternal healthcare institutions distribute accoucheurs to work in units such as emergency department, general wards, intensive care units and operating theatre units, but not in maternity units, despite being trained to work there. Folami (2017:2015) stated that the distribution of accoucheurs to such technical areas might be because of the fact that such roles appear more congruent with the masculine role. Working in such areas seems to decrease the extent of gender strain from accoucheurs because less physical and intimate care is expected from these accoucheurs in these areas (Folami 2017:215). This distribution of accoucheurs based on their gender in the maternal healthcare institutions impacts negatively the training of student accoucheurs as most women lack exposure to the care rendered by accoucheurs, except those who were delivered by accoucheurs in their previous pregnancies. In the study conducted by Duman (2012), some women reported that they did not mind the care provided by male student nurses in maternity wards if everything went well. However, these views were contrary to the study conducted by Mthombeni, Maputle and Khoza (2018) that women perceived that nursing is a profession suitable for women and male midwives cannot work at maternity services because they would not wish to be attended by student accoucheurs during pregnancy and labour. Hence, most of them become resistant towards student accoucheurs during their clinical placement in maternal healthcare units. Nursing institutions should have a natural interest in ensuring that gender bias and stereotypes are minimised in nursing institutions in order to provide equitable learning for all students as well as in creating a nursing workforce that reflects greater gender equality in the nursing profession (Folami 2017:215).

## Problem statement

Over the years gender inequality has been an area of concern in the training of student accoucheurs at the maternal healthcare institutions in the Free State province. This has led to a limited number of student accoucheurs completing their training within record time. The statistics revealed that in 2014, there were 53 student accoucheurs registered and 36 completed; in 2015 there were 29 registered and 20 completed; in 2016, 50 registered but 30 completed, and in 2017, 43 registered but only 28 completed the course (SANC Statistics 2017:1). Gender inequality in maternal healthcare institutions does not only exist in South Africa or the Free State province only but is a universal problem in the training of student accoucheurs (Bwalya et al. 2015:44). Student accoucheurs are faced with various challenges during their clinical placement in maternal healthcare institutions in the Free State province. These challenges include, amongst others, resistance, rejection and discrimination by pregnant women because of gender inequality when placed in maternal healthcare institutions for their maternal health clinical practice. Furthermore, Mthombeni and Phaladi-Digamela (2015:50) revealed that for a student accoucheur to complete the programme, it is expected that the theoretical and practical learning outcomes are achieved. Unfortunately, the rejection, discrimination and resistance they face in maternity units from pregnant women cause barriers in their training. This could lead to non-completion of their required training objectives in midwifery. Therefore, incomplete practical training for student accoucheurs delays their registration as accoucheurs because they would not qualify according to the SANC requirements. Moreover, gender inequality in maternal healthcare institutions creates problems for student accoucheurs who are expected to nurse, examine and conduct deliveries as part of their learning objectives as most women object to maternal healthcare services rendered by student accoucheurs. This creates an academic gap for student accoucheurs’ training. Furthermore, Mthombeni and Phaladi-Digamela (2015:50) reiterated that over the years, student accoucheurs would pass in the theory component of midwifery training and fail in the practical, leading to delays in completing their training course and increased attrition rate. Thus, the present study intended to explore and describe the perceptions of gender inequality on the training of student accoucheurs in the Free State maternal healthcare institutions.

## Research methodology

### Design

An exploratory, descriptive, qualitative research design was undertaken in this study. An explorative design allowed the researcher to identify key issues and variables regarding the perceptions of gender inequality on the training of student accoucheurs (Polit & Beck 2017:780). The descriptive design was used to investigate the ‘who, what and where’ aspects of events (the perceptions of student accoucheurs regarding gender inequality in midwifery training) (Polit & Beck 2017:780). The description of student accoucheurs’ own verbatim responses regarding their perceptions of gender inequality towards their training in maternal healthcare institutions provided a picture of the situation as it was naturally happening, without the manipulation of variables.

### Sample and sampling technique

Only the facilities that had student accoucheurs at the time of the study were purposively selected. The study population consisted of fourth-year student accoucheurs between 22 and 24 years of age who were studying for a Diploma in Nursing (General, Community, Psychiatry) and Accoucheurs (R. 425) at the Free State School of Nursing. The participants were purposively sampled. According to Polit and Beck (2017:691), a qualitative research has no direct fixed sample size; it is driven by the information needs of the study. The sample size was determined by data saturation, which was reached after the eighth focus group discussion, with two more additional focus group discussions to confirm the point of data saturation. Brink, Van Der Walt and Van Rensburg (2017:141) indicated that data saturation is a point at which no new data emerge during data collection. The researcher conducted 10 focus group discussions. Each focus group discussion consisted of four participants; thus, 40 student accoucheurs participated in the study.

### Data collection

Data were collected in November 2017 for a period of 4 weeks from student accoucheurs placed for clinical practice at the Free State maternal healthcare institutions in the Free State province, South Africa. The data collection interview guide was pre-tested with one focus group consisting of three student accoucheurs for its reliability, and no changes were found necessary to be further effected on its usage. Neither the data collected during the pre-testing phase nor the participants were included in the main study. Participants were given the study information letters to read and those who volunteered to participate were given consent forms to sign before the commencement of the study. Ten focus group discussions consisting of four student accoucheurs in each group lasting for 30–45 min were conducted to the point of data saturation, and two further focus group discussions were conducted to confirm data saturation (Creswell 2014:8). Focus-group discussions were conducted in English, which was the preferred language of all participants, most probably because English was the medium of instruction in the Free State province nursing institutions. The focus groups and study participants were assigned codes to ensure confidentiality and anonymity. The main question asked was, ‘[i]n your opinion, what are your perceptions regarding gender inequality in your training as student accoucheurs at the Free State maternal health care institutions?’ This was followed by probing questions to ensure clarity of the participants’ responses. Field notes and voice recorders were used during discussions to capture participants’ actual verbatim responses. To ensure confidentiality, data were stored on a computer with password-protected and in a locked office of the researcher. All focus group discussions were facilitated by the lead researcher (Researcher 1), who is a nurse educator and has prior experience in facilitating focus group discussions.

### Data analysis

Data were analysed verbatim. Tesch’s eight steps of data analysis were used to analyse the data (Creswell 2014:8). Focus group discussions were transcribed verbatim and analysed by the researcher. This included reading the transcripts, comparing them with the audio-taped focus group discussions and using the field notes to confirm their information. The researcher read and reread the transcripts to identify and fully understand the underlying meaning, then selected the most interesting and informative discussions and made notes on the margins of the transcribed focus group discussions. The process was repeated for all the discussions. Similar topics were then clustered together under topics and from these topics the researcher formed themes and subthemes. This was followed by two research co-authors double-checking and confirming the data separately, followed by the identified themes being discussed and mutually agreed upon amongst the three co-authors. Merging themes and sub-themes were identified and confirmed by the researcher and the co-authors.

### Trustworthiness

Polit and Beck (2017:540) defined trustworthiness as the quality, authenticity and the truth of the research finding. The four criteria of credibility, transferability, dependability and confirmability were applied to ensure trustworthiness of qualitative data as established by Lincoln and Guba (1985:301). Credibility was ensured by prolonged engagement. Polit and Beck (2017:561) stated that it is important to invest sufficient time collecting data in order to have an in-depth understanding of the participants under study. Therefore, the researcher spent 30–45 min with each focus group. Furthermore, the researcher ensured member checking, through deliberate probing, to ensure that participants’ meanings were clearly understood to ensure credibility. In order to ensure transferability, information-rich participants were purposively sampled. A comprehensive description of the data included verbatim quotations. The research process was systematically reported so that other researchers could test its applicability in other contexts. Dependability refers to the stability and solidity of data over time (Polit & Beck 2017:559). Thus, the researcher asked the same questions to all participants in order to be consistent. Confirmability was safeguarded through the use of an independent coder, member checking and audit trail.

## Study findings

The total number of student accoucheurs who participated in the research study was 40. All participants (P) were black people, single men between 22 and 24 years of age who were in their fourth-year level of training and were placed for a period of 4 weeks at the Free State maternal healthcare institutions in November 2017. The three major themes and nine sub-themes that emerged formed an important part of common views and perceptions of all the participants. These were synthesised under the following nine sub-themes: midwifery students’ registers, midwifery clinical hours, pregnant women’s rights, student accoucheurs’ rights of midwifery nursing practice, nursing personnel hierarchy, midwifery lecturers, women’s exposure to accoucheurs, long-term goal strategy and breaking midwifery norms. Three major themes were identified as follows: meeting the training requirements in midwifery, women’s autonomy in a choice of healthcare provider versus student accoucheurs’ autonomy to be trained in midwifery and staff establishment to render maternal healthcare.

### Theme 1: Meeting the training requirements in midwifery

The participants expressed strong emotional difficulty in getting maternal cases to enter into their midwifery registers. This was based on gender inequality they had experienced and witnessed since the start of their midwifery training. They perceived their female counterparts as being favoured as they did not experience any difficulty in obtaining maternal cases for their registers. They experienced ambivalence in their feelings:

‘We are experiencing difficulty in getting maternal cases for our registers due to the fact that women refuse to be assisted by us, this is not the case with our female colleagues as they do not have such a problem … eh …, hence at times we have to come even at night outside our scheduled shifts to try and get cases to complete our registers.’ (P5, male, 23 years old)

Other participants felt that they had to work double the hours required by the SANC for midwifery discipline because they had to come even on Saturdays or Sundays, including at night and holidays in order to get maternal cases for their registers. This was captured verbatim, as in the following quote:

‘We put way more hours in midwifery than what is prescribed by the SANC; we work seven days a week and at times even at night just to get cases for our registers as most women tend to resist and refuse to be delivered by us, this can be exhausting at times.’ (P6, male, 22 years old)

### Theme 2: Women’s autonomy in a choice of healthcare provider versus student accoucheurs’ autonomy to practice

Most of the participants during focus group discussions stated that some women indicated to them that if they were given an opportunity to choose the midwife, they would prefer to be assisted by a female midwife. Some student accoucheurs also confirmed this during focus group discussions that most women prefer to be assisted by female midwives over them. This is what they had to say:

‘One of the women I was taking care of stated that if she had to exercise her right to choose between a female and a male midwife … eh … she would definitely prefer to be assisted by a female midwife.’ (P4, male, 24 years old)

Participants expressed a feeling of being rejected and being unwanted by pregnant women in maternity units based on their rights as patients. Some participants linked women’s rights as being influenced by gender inequality in maternal healthcare institutions as they inferred that there were no accoucheurs amongst qualified practising staff in all the maternal healthcare units. Hence, most student accoucheurs felt that they were not welcomed in the maternity units by women as it was clear to them that female midwives were women’s preferred choice. One participant reiterated this:

‘I want to be assisted by a sister … please go call the sister for me.’ (P5, male, 23 years old)

Participants revealed that they too also had rights to be trained and practise midwifery. They argued that their course includes practice and competence in all disciplines including midwifery. This was what participants had to say:

‘We have rights to be trained as accoucheurs, the comprehensive Diploma in Nurse (General, community, Psychiatry) and Midwife/Accoucheur (R.425) require us to be competent in all disciplines.’ (P7, male, 22 years old)

‘We need to be placed and practise midwifery skills in maternity units using pregnant women to be declared competent accoucheurs. This is what is required by the course we are doing.’ (P1, male, 23 years old)

### Theme 3: Staff establishment to render maternal healthcare

The discussion outcomes with the participants indicated that most of maternal healthcare institutions were manned by female midwives. The participants confessed that the nursing personnel hierarchy from top management to midwives in these institutions had no gender equality when it comes to staff distribution in the maternal healthcare units. Participants expressed their sentiments as follows:

‘All staff members in these units are females, trained accoucheurs are not placed in maternity units despite staff shortages in maternity units… Woman only see a male figure during Doctor’s rounds or when community service nurses are placed there or when we are placed there for few weeks.’ (P3, male, 24 years old)

‘Eh … most women told me that they have only seen Doctors and female midwives assisting them in most of the time, …. We only come once in a while in this ward, hence they are reluctant to be assisted by us.’ (P2, male, 23 years old)

Another participant narrated this:

‘… Indeed, in all the units I have been allocated to I only worked with female midwives, even the Unit Managers in all units where I was placed from third year to fourth year level were all females. I only used to see accoucheurs in other units within the institutions.’ (P7, male, 22 years old)

Some of the participants inferred that all of their theoretical and clinical lecturers teaching midwifery at the Free State School of Nursing were women. Most of the participants expressed their concern about the existence of gender inequality at training institutions. They associated their rejection and resistance of women in maternal healthcare units with the notion that even during clinical accompaniment, women only see female lecturers and no male lecturers.

This was clear in one of the participants’ excerpts:

‘All of my lecturers both teaching midwifery theory and clinical practice are females.’ (P7, male, 22 years old)

Another participants stated this:

‘Most of women have noticed that all lecturers coming to do clinical accompaniment were females and that there were no male lecturers.’ (P8, male, 23 years old)

Participants expressed their concern regarding women’s lack of exposure to services rendered by accoucheurs at the maternity units. Some participants indicated that the absence of accoucheurs working in maternity units deprives women of the opportunity to be nursed by accoucheurs, promoting gender inequality in these units. Moreover, it continues to contribute to the rejection and resentment of student accoucheurs by women during their clinical placement in maternal healthcare institutions. One participant stated this:

‘The absence of accoucheurs in maternity units deprives women from the care rendered by accoucheurs; this also has negative impact on our training as women only experience the services rendered by males during our short period of clinical placement, hence most refuse to be assisted by us.’ (P5, male, 23 years old)

However, some student accoucheurs were concerned that the issue of gender inequality in maternal healthcare institutions would negatively disadvantage them in achieving their dreams. Most student accoucheurs professed that they would like to study midwifery discipline further on completion of their training to become advanced accoucheurs, and pursue master’s degree in midwifery. This was some of their utterances:

‘My long term goal is to become an advance accoucheur.’ (P4, male, 22 years old)

‘When I complete my basic diploma, I would like to further my studies focusing mostly in midwifery. My aim is to obtain a master’s degree in midwifery.’ (P8, male, 23 years old)

Some participants uttered that they were prepared to break the norm by requesting their nursing managers to place them in maternal healthcare units upon completion of their course. This was viewed as an opportunity to neutralise the existing gender inequality witnessed during their clinical placement at the maternal healthcare institutions. The following statement emanated from one of the focus group discussions:

‘I am prepared to request the Nursing Manager to place me in maternity unit and to consider placing more accoucheurs in maternity units as this will give me an opportunity to break the norm that midwifery is a female-dominated discipline… and above all to assist in neutralising gender stereotype occurring in these units.’ (P7, male, 22 years old)

### Ethical consideration

Ethical clearance to conduct the study was obtained from the Research Ethics Committee of the Durban University of Technology (IREC Number: 10/17), followed by a request and approval from the Free State Department of Health, Free State District Managers and Free State School of Nursing. Participants were required to have adequate information about the research, comprehend that information and have the choice to consent or decline participation voluntarily (Polit & Beck 2017:521). To ensure this, verbal and written information letters were given to the participants before participating in the research study. All participants signed the consent form to participate in the study, without any coercion.

## Discussion of the study findings

Student accoucheurs implied that they were faced with great challenges during their midwifery clinical practice in the Free State maternal healthcare institutions. This was found to be problematic by student accoucheurs, which may contribute to the perceived gender inequality in this discipline because of various factors, including rejection and discrimination by women. Mohamed (2013:2812) also agreed with the notion that student accoucheurs experience rejection from women in maternity units, leading to delays in meeting the required training objectives. The disadvantage faced by student accoucheurs because of their gender may lead to decreased number of accoucheurs throughput registered in this programme because of the failure in meeting the required midwifery training objectives.

According to SANC Regulation (R 425 of 1985), student nurses registered in this programme should conform to the prescribed scope of practice of student midwives and achieve all clinical competencies prescribed by SANC to be registered as accoucheurs or midwives. Most student accoucheurs tend to work double the shifts and more hours hoping to get women who might be kind enough to allow them to be assisted in antenatal, labour and postnatal care. This could be emotionally, psychologically and, most importantly, physically exhausting to students (Smith et al. 2018:1). The continuation of these stress-inducing gender inequality stereotypes may lead to student accoucheurs losing interest and motivation in the programme. Tzeng et al. (2009:1) revealed that male students faced more gender-based stress than their female counterparts. Hence, most student accoucheurs corroborated with this view that incomplete midwifery registers tend to make them feel sidelined, helpless, unappreciated and left alone to fend for themselves with little support in their training because of their gender.

According to Chan, Chan and Tse (2014:301), gender preferences and barriers between male nurses and female patients have a significant negative impact on the delivery of intimate care by male nurses in maternity units. Meanwhile, student accoucheurs continue to experience difficulties in meeting the training objectives in maternal healthcare institutions. Student accoucheurs attested the notion by stating that some women mentioned that they prefer to be assisted by female midwives. Although some women (10%) preferred to be delivered by student accoucheurs, stating that they are more caring and supporting, the majority of women (90%) still preferred midwives (Duman 2012:3). Therefore, it is evident that there is still an association between gender and midwife preference amongst women regarding the choice of their preferred carer (Shavai & Chinamasa 2015:169).

Women’s autonomy is viewed as the capacity and freedom to act independently, encompassing women’s ability to formulate strategic choices, control resources and participate in decision-making (Ramesh 2016:2). This emanated from the group discussion that most of the participants felt unwelcomed in maternal healthcare institutions by women. Therefore, most participants stated that women were exercising their autonomy in choosing the gender of the midwife they prefer to assist them. This has a negative impact on the training of student accoucheurs in midwifery training. This is supported by Cook and Loomis (2012:158) who stated that most women plan for their pregnancy, type of preferred delivery, place and the midwife they would like to assist them during childbirth. Therefore, their choices should be respected by the healthcare providers, including the student accoucheurs. The autonomy of women in choosing their preferred maternal healthcare provider including the gender of midwife contributes to gender inequality in the training of student accoucheurs as they feel rejected in rendering maternal healthcare to women.

It has been noted in various healthcare institutions that gender inequality in the workplace is a major challenge in most maternal healthcare institutions (Newman 2014:1). This gender inequality amongst the healthcare workforce deprives student accoucheurs the opportunity to work with qualified accoucheurs as their role models who will support, guide and mentor them in midwifery nursing. Buthelezi et al. (2015:7) agreed that more male accoucheurs should take the lead to motivate their younger male nurses. Gender inequalities are the system’s inefficiencies that contribute to clogged health worker educational pipelines, recruitment bottlenecks, attrition and misdistributions of staff in the institutions (Newman 2014:3). This was attested by the student accoucheurs who indicated that in the maternal healthcare institutions where they were placed, none of the accoucheurs were either unit managers or assistant nursing managers. Within the hierarchical structures of the maternal healthcare institutions, gender equality was not considered; hence, all nursing managers were women. Ofori (2007:17) revealed that most men in nursing tend to rise to the top of their occupations more quickly than their female counterparts in other disciplines of nursing, but not in midwifery discipline. Therefore, student accoucheurs do not have an accoucheur nurse manager to look upon them for encouragement and support. In conclusion, it is important that attention should be given to gender equality when appointing nursing personnel in key positions to fill managerial hierarchical structures of these maternal healthcare institutions. This will neutralise gender inequality and accommodate support, mentorship and guidance of student accoucheurs when they are placed in maternal healthcare units where there are accoucheurs within the healthcare workforce.

The participants corroborated that in the maternal healthcare institutions there were only female midwives and there were no accoucheurs to whom they could look up to as role models for emotional support and courage in midwifery nursing. Chinkhata (2016:9) recommended that where applicable institutions should ensure availability of accoucheurs to act as role models both in college as lecturers and in hospitals. Although there were a significant number of qualified accoucheurs in the institutions, all of them were placed in areas such as general wards and emergency departments. According to Yi and Keogh (2016:96), the Nursing and Midwifery Board of Ireland revealed that there were about 10% male nurses amongst all registered nurses in 2012. The notion was concurred by Folami (2017:215) that male nurses were usually moved to technical areas, such as operating theatres and emergency rooms, because such areas appeared more congruent with the masculine role of men. Furthermore, student accoucheurs attested that there were no male lecturers teaching either theory or clinical midwifery discipline in the Free State School of Nursing. This was seen as a lack of male role models in the training institutions who could give student accoucheurs emotional support, guidance and share experiences with them on how they overcame the challenges. Achora (2016:27) agreed with the notion by stating that there is a need for institutional support for male student nurses in midwifery practice. Therefore, it is believed that, indeed, gender inequality exists in the maternal healthcare institutions as well as in the training institutions for nurses in Free State province.

Student accoucheurs reported that most women affirmed that they are not used to the maternal healthcare services rendered by men in the maternity units. Cook and Loomis (2012:158) confirmed that in most rural non-training maternal healthcare institutions, doctors are the only male figures attending to pregnant women. This was an affirmation that, indeed, gender inequality has a negative impact on midwifery clinical placement of student accoucheurs in the maternal healthcare institutions. Most student accoucheurs stated that amongst all four disciplines they were exposed to during their training, they would prefer midwifery despite the challenges they faced during their training. Participants further preferred to advance their studies in Advanced Midwifery on completion of their training to neutralise gender inequality in midwifery, and act as role models to the student accoucheurs. Furthermore, some student accoucheurs preferred to practise as accoucheurs rather than professional nurses working in general units. The Jamaican Observer on 08 May 2018 indicated that since the 19th century feminisation of midwifery career, men have embraced the status quo (Champbell 2018). Therefore, the suggestion is to attract more men into midwifery by re-orientating the public that midwifery is not a career for women only (Champbell 2018). This will also lead them to break the boundaries of gender inequality stereotypes existing in maternal healthcare institutions.

## Limitation of the study

The findings of this study cannot be generalised to other settings because of the limited and small sample size. Women were also not included in the study as the information from them could have enriched the study findings.

## Recommendations

The following recommendations are made:

- Gender equality in maternal healthcare institutions should be considered in the Free State Department of Health policies. Service delivery guidelines are to be implemented in all maternal healthcare institutions where student accoucheurs are placed to assist them with challenges they face in maternity units.
- Nurse managers should ensure that willing and passionate accoucheurs form part of the staff establishment in the maternity care units in order to familiarise women with nursing care rendered by student accoucheurs.
- Nurses training institutions should strengthen their curriculum to address gender inequality in midwifery to empower student accoucheurs with skills and knowledge and offer support during their clinical placement in maternal healthcare institutions.
- Maternal healthcare institutions should provide education regarding the importance of availability and training of student accoucheurs, including their roles to pregnant women to accustom them with gender equity in midwifery nursing including improvement of their acceptance.
- Health directorates should consider recruiting, appointing and placing or promoting existing male nurse managers who are trained in midwifery to manage maternity units in order to improve gender equity and role modelling of student accoucheurs in midwifery discipline.

## Conclusion

This study confirmed that gender discrimination contributes negatively to the training of student accoucheurs in the Free State maternal healthcare institutions. These could be overcome if health directorates, nurse managers and training institutions take into consideration gender inequality in the nursing workforce during revision or development of policies, curriculum and appointment of staff members.
